# Modelling of the Behaviour of *Salmonella enterica serovar* Reading on Commercial Fresh-Cut Iceberg Lettuce Stored at Different Temperatures

**DOI:** 10.3390/foods9070946

**Published:** 2020-07-17

**Authors:** Fatih Tarlak, Gro Johannessen, Isabel Bascón Villegas, Araceli Bolívar, Guiomar Denisse Posada-Izquierdo, Fernando Pérez-Rodríguez

**Affiliations:** 1 Department of Nutrition and Dietetics, Istanbul Gedik University, 34876 Istanbul, Turkey; ftarlak@gtu.edu.tr; 2 Department of Animal Health and Food Safety, Norwegian Veterinary Institute, P.O.Box 750 Sentrum, 0106 Oslo, Norway; gro.johannessen@vetinst.no; 3 Department of Food Science and Technology, Faculty of Veterinary, Agrifood Campus of International Excellence (ceiA3), University of Cordoba, 14014 Córdoba, Spain; q12bavii@gmail.com (I.B.V.); t12bocaa@uco.es (A.B.); guionisse@hotmail.com (G.D.P.-I.)

**Keywords:** Weibull model, predictive microbiology, food safety, modified atmosphere packaging, *Salmonella* Reading

## Abstract

The aim of this study was to model the growth and survival behaviour of *Salmonella* Reading and endogenous lactic acid bacteria on fresh pre-cut iceberg lettuce stored under modified atmosphere packaging for 10 days at different temperatures (4, 8 and 15 °C). The Baranyi and Weibull models were satisfactorily fitted to describe microbial growth and survival behaviour, respectively. Results indicated that lactic acid bacteria (LAB) could grow at all storage temperatures, while *S*. Reading grew only at 15 °C. Specific growth rate values (*μ_max_*) for LAB ranged between 0.080 and 0.168 h^−1^ corresponding to the temperatures 4 and 15 °C while for *S*. Reading at 15 °C, *μ_max_* = 0.056 h^−1^. This result was compared with published predictive microbiology models for other *Salmonella* serovars in leafy greens, revealing that predictions from specific models could be valid for such a temperature, provided they were developed specifically in lettuce regardless of the type of serovars inoculated. The parameter delta obtained from the Weibull model for the pathogen was found to be 16.03 and 18.81 for 4 and 8 °C, respectively, indicating that the pathogen underwent larger reduction levels at lower temperatures (2.8 log_10_ decrease at 4 °C). These data suggest that this *Salmonella* serovar is especially sensitive to low temperatures, under the assayed conditions, while showcasing that a correct refrigeration could be an effective measure to control microbial risk in commercial packaged lettuce. Finally, the microbiological data and models from this study will be useful to consider more specifically the behaviour of *S*. Reading during transport and storage of fresh-cut lettuce, elucidating the contribution of this serovar to the risk by *Salmonella* in leafy green products.

## 1. Introduction

In recent years, the technological progress in food industry and consumer demand for a healthy lifestyle have led to a rise in the production and consumption of fresh produce, specially minimally processed pre-cut fruits and vegetables. In this regard, the fresh-cut industry has experienced a faster growth rate than other sectors of the fruit and vegetable market [1]. Particularly, packaged lettuce accounts for about 50% the fresh-cut market volume [2]. At the same time, there has been an increase in the number of foodborne outbreaks associated with such food commodities. The main challenge is that fresh-cut products are generally ready-to-eat (RTE) and consumed raw without any heat treatment or other microbial inactivation method applied prior to consumption that eliminates fully foodborne pathogens.

Contamination of vegetables in the field might be due to, among other factors, faecally contaminated irrigation water being a potential source of enteric pathogens [3]. The use of chlorine in post-harvest washing and modified atmosphere packaging (MAP), together with refrigerated storage, are the most typical preservation technologies used to ensure the food safety and quality of fresh-cut fruits and vegetables [4]. Despite that, washing water, cutting, packaging and food handlers can be sources of microbial contamination [5,6]. Indeed, the process of post-harvest washing can potentially lead to cross-contamination events if appropriate concentrations of sanitizer are not maintained [7].

According to the latest European Food Safety Authority (EFSA) and European Centre for Disease Prevention and Control (ECDC) EFSA/ECDC report on zoonoses and zoonotic agents, *Salmonella enterica* is the second most common cause of foodborne outbreaks after *Campylobacter* in the European Union (EU) [8]. In addition, *Salmonella* has been reported to be the second largest contributor to produce-associated outbreaks both in the United Stated (US) and the EU [9]. Particularly, leafy greens eaten raw as salads have been identified as the most frequent food commodity of non-animal origin implicated in *Salmonella* human infections in the EU [10]. *S*. Enteritidis is the predominant serovar in human cases acquired in the EU [8]. The most reported *Salmonella* serovars causing leafy vegetable-associated outbreaks in the US during 1973–2012 were Enteritidis and Typhimurium, followed by Javiana and Newport [11]. In the past, the serovar Reading has been uncommonly associated with human illness and rarely involved in foodborne outbreak. However, during the last years there has been a change in this trend with a rise of reported cases of *S*. Reading associated with different contaminated food items [12]. Lienemann et al. [13] investigated a nationwide outbreak caused by *S*. Newport and *S*. Reading in Finland and suggested pre-cut iceberg lettuce as the source of the outbreak. Robertson et al. [14] isolated *S*. Reading from processing water used in the production of mung bean sprouts. More recently, multidrug-resistant *S*. Reading has caused a multistate outbreak linked to raw turkey products in the US during 2017–2019, involving 358 cases in 42 states [15]. 

Predictive microbiology models are used to predict bacterial kinetics in specific food environments. Over the last decades, mathematical models have become a valuable information source to conduct risk assessment studies and support risk managers concerning microbial food safety and quality issues. A number of predictive models are available for *Salmonella* on leafy greens [16,17,18,19,20,21]. These models have been mostly developed for the serovars Enteritidis and Typhimurium, and at present, the individual survival and growth behaviour of *S.* Reading has not been reported in any food. 

The objective of this study was to assess and model the growth and survival of *S.* Reading on fresh-cut RTE iceberg lettuce stored at different temperatures (4, 8 and 15 °C) for 10 days.

## 2. Materials and Methods 

### 2.1. Sample Preparation and Microbiological Analysis

Fresh-cut RTE iceberg lettuce (*Lactuca sativa* L.) in bags (200 g) under MAP was obtained on the day of production from a local lettuce processing plant located in the Oslo area (Norway). The bags were collected at the processing plant and immediately transported to the laboratory for inoculation and initial testing. 

#### 2.1.1. Preparation of Inoculum

*Salmonella* Reading VI 51763, previously isolated from spent irrigation water used for sprout production [14], was plated on two blood agar plates (OXOID, Basingstoke, UK) from glycerol stocks kept at −80 °C and incubated at 37 °C for 24 h. A single colony was transferred to nine ml of buffered peptone water (BPW) (OXOID, Basingstoke, UK) and incubated for 24 h at 37 ± 1 °C. After incubation, the culture was serially diluted in peptone saline (PS) (0.1% peptone and 0.85% NaCl). The initial inoculum was quantified by plating 100 μL of the appropriate dilution on blood agar plates in triplicate, which were incubated at 37 ± 1 °C for 18–24 h before enumeration. The concentration of the inoculum varied between log_10_ 4.83 and log_10_ 5.08 cfu/mL.

#### 2.1.2. Inoculation of Lettuce Bags

Lettuce bags were inoculated with 100 μL from the appropriate dilution following a similar protocol to the work by Carrasco et al. [22]. Briefly, 100 μL was inoculated through penetrating an adhesive silicon patch on each lettuce bag, using a 1 mL syringe and 0.6 × 25 mm needles. The area around the patch was marked in order to facilitate subsequent sampling. 

Inoculated and control (non-inoculated) lettuce bags were stored at 4, 8 and 15 °C for 10 days (product use-by date). At storage days 1, 2, 3, 4, 7 and 10, five randomly selected lettuce bags were analysed separately for enumeration of *S*. Reading and endogenous lactic acid bacteria (LAB). Experiments were performed in triplicate for each storage temperature. In addition, control bags were analysed at day 0 and day 10 of storage for enumeration of total aerobic viable count (AVC) and for presence/absence of *Salmonella* spp. 

#### 2.1.3. Bacterial Analyses

*S.* Reading was quantified by homogenizing 25 g of inoculated lettuce in 225 mL BPW with a Stomacher (Stomacher 400 Lab Blender, Seward, UK) for 30 s. Tenfold dilution series of the homogenate was prepared in PS and 100 μL of the appropriate dilutions was plated in parallel on Xylose-Lysine-Desoxycholate agar (XLD) (OXOID) and Brilliance™ *Salmonella* agar (OXOID). The plates were incubated at 37 °C for 24 ± 3 h. Typical colonies were counted, and one typical colony was selected for further confirmation. *S.* Reading was confirmed by testing presumptive colonies on Triple Sugar Iron agar (Difco, MD, USA) and Urea agar-containing agar base (OXOID) with 40% Urea (Sigma-Aldrich, St. Louis, MO, USA) followed by agglutination with omnivalent *Salmonella* antiserum (Enteroclon Anti-Salmonella A-67, omnivalent, Sifin, Berlin, Germany). The same homogenate was used for LAB enumeration following Nordic Committee on Food Analysis (NMKL) no. 140 2nd ed., 2007. Plates were incubated anaerobically at 25 °C for five days. 

The detection of *Salmonella* spp. in control bags was conducted in accordance with the NMKL no. 71 5th ed., 1999. Briefly, a homogenate was prepared as described above and incubated at 37 °C for 24 ± 3 h. An aliquot of 100 μL of the enrichment was transferred to 10 mL Rappaport Vassiliadis Soya Peptone broth (RVS) (OXOID) and incubated for 24 ± 3 h at 41.5 °C, followed by plating of a loopful (10 μL) of broth on XLD agar (OXOID) and Brilliance™ Salmonella agar (OXOID) and incubated as described above. Analysis of AVC in control bags was carried out following the method NMKL no. 86, 5th ed., 2013. Plates were incubated under aerobic conditions for three days at 25 °C. 

### 2.2. Microbial Modelling

#### 2.2.1. Growth Model

The Baranyi model [23] as defined by Dabadé et al. [24] and Den Besten et al. [25] was employed to estimate the growth kinetic parameters of microorganisms on fresh-cut iceberg lettuce stored at isothermal conditions:

(1) $$N\left(t\right)=N_{0}-\frac{\mu_{max}}{\ln\left(10\right)}+A\left(t\right)-\frac{1}{\ln\left(10\right)}\ln\left(1+\frac{e^{\mu_{max}\cdot A\left(t\right)}-1}{10^{\left(N_{max}-N_{0}\right)}}\right)$$

where *t* is the time (h), *N*(*t*) is the number of microorganisms (log_10_ CFU/g) at time *t*, *N*_0_ is the initial number of microorganisms (log_10_ CFU/g), *N_max_* is the maximum number of microorganisms (log_10_ CFU/g) and *μ_max_* is the maximum specific growth rate (h^−1^).

The adjustment function of *A*(*t*) is expressed by Equation (2):

(2) $$A\left(t\right)=t+\frac{1}{v}\ln\left(e^{-vt}+e^{-\mu_{max}\lambda }-e^{\left(-vt-\mu_{max}\lambda \right)}\right)$$

where *λ* is the lag phase duration (h), and *v* is the rate of increase of a limiting substrate, assumed to be equal to *μ_max_*.

The Baranyi model was employed to describe microbiological growth data, and growth parameters were estimated with the NonLinearModel.fit command in the Matlab 8.3.0.532 (R2014a) software (MathWorks Inc., Natick, MA, USA). Determination of starting values in nonlinear regression procedure is a critical step to predict the accurate parameters. Starting values for the parameters *N*_0_ and *N_max_* were selected as the minimum and maximum values of experimental data at each temperature, respectively. The starting value of *μ_max_* was chosen as the highest value among the first derivatives of the natural logarithm of bacterial count with respect to time. Parameter *λ* was obtained from the raw data plot as the time point, when exponential growth started. After several successive iterations in the nonlinear procedure, the starting values were converged to optimum values of the parameters.

#### 2.2.2. Survival Kinetic Model

Survival kinetic data of *S.* Reading on the fresh iceberg lettuce stored at 4 and 8 °C were modelled using Weibull model as described by Mafart et al. [26]:

(3) $$N\left(t\right)=N_{0}-{\left(\frac{t}{delta}\right)}^{p}$$

where *t* is the time (h), *N*_0_ is the concentration of microbial population (log_10_ CFU/g) at time *t*, *delta* is the scale parameter and *p* is shape parameter.

Similar to the Baranyi model, the parameters of the Weibull model were calculated with the NonLinearModel.fit command in the Matlab 8.3.0.532 (R2014a) software (MathWorks Inc., Natick, MA, USA).

#### 2.2.3. Goodness of Fit of the Growth and Survival Models

The goodness of fit of the growth and survival models was evaluated by considering the mean squared error (MSE) and adjusted coefficient of determination (adjusted-R^2^) values using Equations (4) and (5), respectively:

(4) $$MSE=\overset{n}{\underset{i=1}{\sum }}\frac{{\left(observed_{i}-fitted_{i}\right)}^{2}}{n-s}$$

(5) $${\mathrm{adjusted}-R}^{2}=1-\left(\frac{n-1}{n-s}\right)\left(\frac{SSE}{SST}\right)$$

where *observed* is the value obtained in experiments, *fitted* is the value estimated by the model, *n* is the number of observations, *s* is the number of parameters of the model, *SSE* is the sum of squared errors and *SST* is the total sum of squares.

## 3. Results and Discussion

The obtained growth data of LAB on the fresh iceberg lettuce for the temperatures of 4, 8, and 15 °C are presented in Table 1. The initial concentration of LAB on fresh iceberg lettuce at 4, 8 and 15 °C, were 3.10 ± 0.06, 3.51 ± 0.07 and 3.75 ± 0.35 log_10_ CFU/g, respectively. These results are in agreement with the values reported in other studies for the initial contamination of LAB in lettuce [19,27,28,29]. After 10 days of storage, the LAB concentrations on the fresh iceberg lettuce were 5.62 ± 0.17, 7.16 ± 0.34 and 8.36 ± 0.13 log_10_ CFU/g for the temperatures of 4, 8 and 15 °C, respectively (Table 1). In addition, control samples were analysed at day 0 and day 10 of storage for enumeration of AVC and for presence/absence of *Salmonella* spp. The initial concentration of AVC on iceberg lettuce was 4.48 ± 0.58, 4.45 ± 0.57 and 5.35 ± 0.17 log_10_ CFU/g, for the storage temperatures of 4, 8 and 15 °C, respectively. At 10 storage days, the AVC concentration was found to reach 7.57 ± 0.25, 8.85 ± 0.25 and 8.35 ± 0.24 log_10_ CFU/g for temperatures of 4, 8 and 15 °C, respectively. *Salmonella* spp. were not detected in any of the control samples of iceberg lettuce throughout the storage period.

LAB presented growth at all tested temperatures. The *μ_max_* and *λ* values of LAB on fresh iceberg lettuce calculated by the Baranyi model for each storage temperature are given in Table 2. The Baranyi model resulted in values of MSE = 0.132 and adjusted-R^2^ = 0.946. These statistical indices showed that LAB growth on the fresh iceberg lettuce could be described properly by the Baranyi model for the isothermal storage temperatures in the range of 4 and 15 °C (Table 2). *μ_max_* values increased from 0.080 to 0.168 h^−1^ between the temperatures of 4 and 15 °C. In contrast, the *λ* values decreased from 51.1 to 21.9 h with the increasing temperature from 4 to 8 °C, while no lag time (adaptation) was observed for 15 °C.

**Figure 1:**
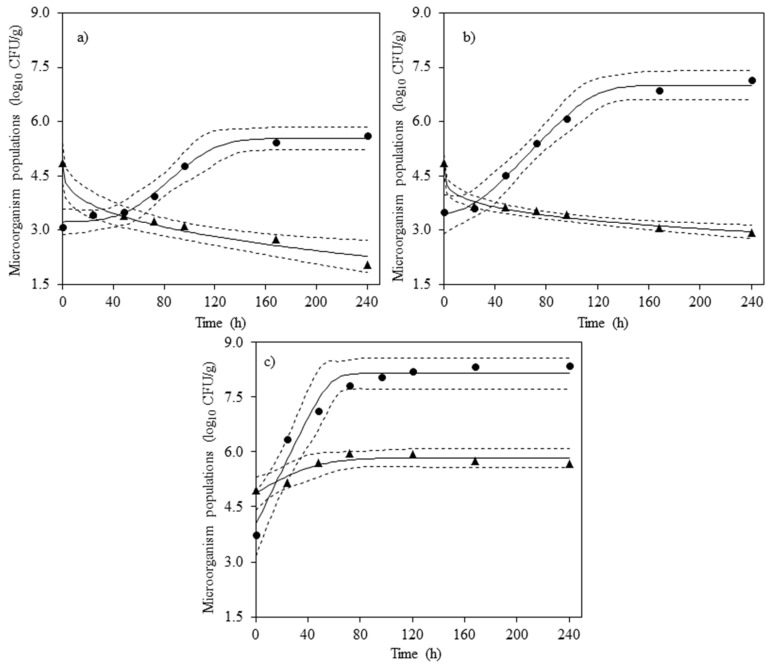
Experimental data of lactic acid bacteria (●) and *Salmonella* Reading (▲) obtained on fresh-cut iceberg lettuce stored at 4 °C (**a**), 8 °C (**b**) and 15 °C (**c**). The solid (—) and dashed (- - -) lines show the fitted values and their confidence bands at 95%, respectively.

The initial *S.* Reading concentration on the inoculated lettuce bags was around 5 log_10_ CFU/g for all storage temperatures (Table 1). At the end of the storage period (10 days), *S.* Reading concentration was 2.06 ± 0.08 and 2.93 ± 0.25 log_10_ CFU/g at 4 and 8 °C, respectively, while at 15 °C the pathogen reached 5.68 ± 0.29 log_10_ CFU/g. These results demonstrated that *S.* Reading exhibited growth on the tested product only at 15 °C. The fitting of the Baranyi model to these data was acceptable as evidenced by the graphical representation in Figure 1 and the value obtained for the goodness of fit indexes (MSE = 0.029 and adjusted-R^2^ = 0.806). The fitted model showed no adaptation time (*λ*) and an *μ_max_* value of 0.056 h^−1^ (Table 2).

The growth kinetics for different *Salmonella* serovars in lettuce reported by other studies were in line with the results for *S.* Reading at 15 °C. However, in several cases, studies demonstrated growth at temperatures of 8 °C or even slightly lower, which was not observed for *S.* Reading in this study. This is probably due to serovar and strain variations with respect to growth temperatures. For instance, Sant’Ana et al. [19] studied the growth of a cocktail of *Salmonella enterica* composed of the serovars Typhimurium and Enteritidis in lettuce packaged under modified atmosphere conditions and incubated between 7 and 30 °C. According to experimental data, all *Samonella* strains were able to grow at 7 °C. Other studies have also shown growth at 5 °C for several *Salmonella* serovars such as Enteritidis, Typhimurium, Anatum, Newport and Saint Paul [20,30]. In turn, Koseki and Isobe [16] did not find growth at 5 °C for the serovars Typhimurium and Enteritidis. The experiments of the work by Koseki and Isobe [16] were also carried out under the same air conditions as those used in the study by Oliveira et al. [30].

Concerning the prediction capacity of existing microbiology models to estimate *S.* Reading kinetics, models predicted growth at lower temperatures than 15 °C, which was the only temperature showing growth in our study. Basically, models reflected what was found in the experimental data (i.e., model domain), thus the model from Sant’Ana et al. [19] and Koseki and Isobe [16] predicted growth at 7 and 10 °C, respectively, while the models obtained by Oliveira et al. [30] and Veys et al. [20] predicted growth at a temperature as low as 5 °C. At 15 °C, model results were in agreement on a practical absence of lag time, while *μ_max_* ranged from 0.05 and 0.08 h^−1^. The best match with the growth rate observed in our work (*μ_max_* = 0.056 h^−1^) was found for the models from Sant’Ana et al. [19] and Koseki and Isobe [16], with values of 0.053 and 0.057 h^−1^, respectively. It is noteworthy that these models were the ones with a more similar minimum growth temperature to our study. For other studies, in which other leafy greens were used, results were much more different. For example, the secondary model developed by Mishra et al. [17] based on data collected from literature for leafy greens including iceberg lettuce predicted a *μ_max_* value at 15 °C of 0.096 h^−1^, while Yoon et al. [21] studying *S.* Typhimurium on fresh-cut cabbage reported *μ_max_* and *λ* values for 15 °C of 0.034 h^−1^ and 10.2 h, respectively. In summing up, the microbiology models developed exclusively with the serovars Enteritidis and Typhimurium in a similar matrix would be the only ones suitable for predicting *S.* Reading growth at 15 °C, and probably at temperatures around this value but not at temperatures near to the minimum growth temperature given the discrepancies obtained for lower temperatures.

With regard to the possible explanation for these similarities between studies and models, it is likely that the relatively high temperature was a larger determinant than other factors (packaging atmosphere, inoculum method, type of lettuce, etc.). From this, it is apparent that the serovar would not be especially relevant under more optimum growth conditions. Nevertheless, it is remarkable that, in our study, *S.* Reading was not able to grow at 8 °C, indicating that this strain could have a reduced growth potential under the conditions studied in comparison to other studies. In this sense, we hypothesize that the endogenous microbiota of lettuce could also play an important role as an inhibitory factor on *Salmonella*, as in our study, LAB was able to grow at all temperatures with a higher growth rate. Nevertheless, more specific research is needed to determine this potential effect from native LAB on *Salmonella*.

The survival data of *S.* Reading on the fresh iceberg lettuce were satisfactorily described using the Weibull model as can be observed in Figure 1, with all experimental data within 95% confidence bands, which was also confirmed by the goodness of fit indexes, with MSE < 0.041 and adjusted-R^2^ > 0.944 (Table 3). The parameter *delta* increased from 16.03 to 18.81 at 4 and 8 °C, respectively, while the parameter *p* slightly decreased from 0.348 to 0.250. Mathematically, the *delta* value can be interpreted as the time needed to reduce the first decimal reduction of the microbial population. Therefore, its increase observed between 4 and 8 reflects a reduction in the die-off rate of the *Salmonella* population. Besides, *p* values were < 1, confirming that the microorganism followed a concave upward curve profile as observed in survival curves (Figure 1). This survival profile is characterized by an asymptotic region, which could involve a resistant and residual population that could remain viable for a longer time despite of the initial die-off rate.

To the best of our knowledge, there are no studies reporting the survival kinetic parameters for *Salmonella* in lettuce during storage, even though some works provided estimates of the logarithmic reductions that could be compared to the ones obtained herein. For instance, the study by Chang and Fang [31], where shredded iceberg lettuce inoculated with *S.* Typhimurium was incubated under aerobic conditions at 4 °C, obtained a reduction of 0.7 ± 0.4 and 0.8 ± 0.3 log_10_ CFU/g for 3 and 7 days, respectively. Whereas, in our study, reductions were higher, with values of 1.7 and 2.3 log_10_ CFU/g for 3 and 7 days, respectively. Besides that, Delbeke et al. [32] observed, after 7 days, reductions of around 1 log_10_ CFU/g of *S.* Thompson in lettuce packaged in passive atmosphere and incubated at 7 °C. In this case, values in our work were also higher, with a reduction of 1.8 log_10_ CFU /g for the temperature of 8 °C. In the light of these data, apart from the possible differences between serovars, it can be suggested that reduced oxygen content (2–4% O_2_) in the packaging atmosphere could enhance the inactivation of *Salmonella* when stored at refrigeration conditions, with log_10_-decreases of 2.8 and 2.0 for 4 and 8 °C, respectively for 10 days of storage. These findings demonstrated that for *S*. Reading, under the typical commercial conditions for cut lettuce (packaged in MAP), an adequate refrigeration temperature may not only inhibit its growth, but reduce drastically the pathogen contamination in the commercialized product.

## 4. Conclusions

The Baranyi model described accurately the growth behaviour of LAB on the fresh iceberg lettuce at different storage temperatures (4, 8 and 15 °C). *S.* Reading could not grow on the fresh iceberg lettuce stored at 4 and 8 °C. The comparison with other studies demonstrated that the microbiology models for lettuce developed in other *Salmonella* serovars could be used for predicting *S.* Reading growth at 15 °C, but not at low temperatures, given the discrepancy in the minimum growth temperatures. Besides that, the survival data of *S.* Reading suggested a low tolerance of this *Salmonella* serovar to the storage temperatures of 4 and 8 °C, with higher reduction levels than those reported in literature. The survival kinetics could be properly reflected by using the Weibull model. Results and predictive microbiology models obtained in this study provide a valuable source of information to consider, in quantitative risk assessment and in predictive analysis, the kinetics of a specific *Salmonella* serovar, *S.* Reading, on commercial fresh iceberg lettuce.

## Figures and Tables

**Table 1:** Experimental data of lactic acid bacteria and *Salmonella* Reading on fresh iceberg lettuce stored at different temperatures for up to 10 days.

		Lactic Acid Bacteria (log_10_ CFU/g) ^a^			*Salmonella* Reading (log_10_ CFU/g) ^a^		
Storage Temperature (°C)		4	8	15	4	8	15
Storage Time (day)	0	3.10 ± 0.06	3.51 ± 0.07	3.75 ± 0.35	4.87 ± 0.04	4.86 ± 0.05	4.95 ± 0.11
	1	3.42 ± 0.18	3.61 ± 0.08	6.34 ± 0.52	3.46 ± 0.20	3.67 ± 0.11	5.16 ± 0.06
	2	3.49 ± 0.19	4.53 ± 0.18	7.13 ± 0.69	3.39 ± 0.21	3.62 ± 0.15	5.70 ± 0.13
	3	3.96 ± 0.50	5.42 ± 0.18	7.82 ± 0.05	3.25 ± 0.16	3.53 ± 0.14	5.95 ± 0.40
	4	4.78 ± 0.64	6.08 ± 0.01	8.04 ± 0.05	3.13 ± 012	3.42 ± 0.13	-
	7	5.44 ± 0.73	6.85 ± 0.62	8.19 ± 0.13	2.74 ± 035	3.07 ± 0.30	5.74 ± 0.22
	10	5.62 ± 0.17	7.16 ± 0.34	8.36 ± 0.13	2.06 ± 0.08	2.93 ± 0.25	5.68 ± 0.29

^a^ Average value ± Standard deviation (*n* = 3).

**Table 2:** Estimated growth parameters for the different storage temperatures.

Bacteria	Temp. (°C)	Parameters ^a^				MSE ^b^	R^2 c^
		*N*_0_ (log_10_ CFU/g)	*N_max_* (log_10_ CFU/g)	*μ_max_* (h^−1^)	*λ* (h)		
LAB	4	3.23 ± 0.110	5.54 ± 0.102	0.080 ± 0.018	51.1 ± 9.5	0.020	0.981
	8	3.45 ± 0.168	7.00 ± 0.125	0.087 ± 0.012	21.9 ± 9.4	0.031	0.986
	15	4.04 ± 0.335	8.14 ± 0.166	0.168 ± 0.028	-	0.132	0.946
*S.* Reading	15	4.87 ± 0.162	5.83 ± 0.94	0.056 ± 0.005	-	0.029	0.806

^a^ Estimated parameters ± Standard error. ^b^ Mean squared error (MSE). ^c^ Adjusted coefficient of determination (adjusted-R^2^).

**Table 3:** Estimated parameters for the survival kinetic model of Weibull.

Bacteria	Temp. (°C)	Parameters ^a^			MSE ^b^	R^2 c^
		*N*_0_ (log_10_ CFU/g)	*delta*	*P*		
*Salmonella* Reading	4	4.84 ± 0.201	16.03 ± 10.5	0.348 ± 0.075	0.041	0.944
	8	4.85 ± 0.087	18.81 ± 7.81	0.250 ± 0.036	0.007	0.981

^a^ Estimated parameters ± standard error. ^b^ Mean squared error (MSE). ^c^ Adjusted coefficient of determination (adjusted-R^2^).
